# Optimizing the manure substitution rate based on phosphorus fertilizer to enhance soil phosphorus turnover and root uptake in pepper (*Capsicum*)

**DOI:** 10.3389/fpls.2024.1356861

**Published:** 2024-03-05

**Authors:** Kai Sun, Yutao Cui, Linglulu Sun, Bingli Wei, Yuan Wang, Shunjin Li, Chengxiang Zhou, Yixia Wang, Wei Zhang

**Affiliations:** ^1^ College of Resources and Environment, Interdisciplinary Research Center for Agriculture Green Development in Yangtze River Basin, Academy of Agricultural Sciences, Institute of Innovation and Entrepreneurship Hanhong College, Southwest University, Chongqing, China; ^2^ Key Laboratory of Green and Low-carbon Agriculture in Southwest Mountain, Ministry of Agriculture and Rural Affairs, Chongqing, China; ^3^ State Key Laboratory of Nutrient Use and Management, College of Resources and Environmental Sciences, China Agricultural University, Beijing, China

**Keywords:** manure substitution, phosphorus, root traits, phosphorus fraction, pepper

## Abstract

**Introduction:**

In contemporary agriculture, the substitution of manure for chemical fertilizer based on phosphorus (P) input in vegetable production has led to a significant reduction in P fertilizer application rates, while, the effect of manure substitution rates on soil P transformation and uptake by root remain unclear.

**Methods:**

This research conducts a pot experiment with varying manure substitution rates (0%, 10%, 20%, 30%, 40%, 50%, 75% and 100%) based on P nutrient content to elucidate the mechanisms through which manure substitution affects P uptake in pepper.

**Results and discussion:**

The result showed that shoot and root biomass of pepper gradually increased as manure substitution rate from 10% to 40%, and then gradually decreased with further increases in the substitution rate. Soil alkaline phosphatase activity and arbuscular mycorrhizal (AM) colonization gradually increased with manure substitution rates improvement. Specifically, when the substitution rate reached 30%–40%, the alkaline phosphatase activity increased by 24.5%–33.8% compared to the fertilizer treatment. In contrast, phytase activity and the relative expression of phosphate transporter protein genes in the root system was declined after peaking at 30% manure substitution. Additionally, soil available P remained moderate under 30%–40% substitution rate, which was reduced by 8.6%–10.2% compared to that in chemical fertilizer treatment, while microbial biomass P was comparable. In the current study, soil labile P similar to or even higher than that in chemical fertilizer treatment when the substitution rate was ≤40%. Correlation heatmaps demonstrated a significant and positive relationship between soil available P and factors related to labile P and moderately labile P.

**Conclusion:**

This finding suggested that substituting 30%–40% of chemical P with manure can effectively enhance root length, AM colonization, soil enzyme activity, soil labile P, and consequently improve P uptake in pepper. These findings provide valuable insights for future organic agricultural practices that prioritize P supply, aiming to standardize organic P management in farmland and achieve high crop yields and maintain soil health.

## 1 Introduction

Phosphorus (P) is a vital element in vegetable production, playing a crucial role in promoting root growth and increasing yields (Li et al., 2016; Wang et al., 2019; Bindraban et al., 2020). The demand for P in crop production in China led to the rapid growth of the P fertilizer industry since the late 20th century (Li et al., 2015). However, the extensive exploitation of P resources within a short timeframe has resulted in a depletion of these resources (Liu et al., 2020b; Gong et al., 2022). Notably, China annually produces approximately 5.7 billion tons of organic materials, equivalent to about 13 million tons of P_2_O_5_ (Niu and Ju, 2017), which are increasingly being utilized as substitutes for P in agriculture production, highlighting the importance of improving P recycling in agricultural waste for sustainable agriculture green development (Yi et al., 2021).

Pepper is a globally significant vegetable with high nutritional value, providing essential components such as capsaicin, carotene, and vitamins, which are crucial for a balanced diet (Baenas et al., 2019). China is a major producer and consumer of peppers, accounting for around 40% of the global planted area in 2021 (Food and Agriculture Organization of the United Nations, 2021, http://www.fao.org/faostat/en/). However, intensive vegetable production often leads to the accumulation of excess soil P and increased environmental stress due to the use of chemical P fertilizer. In this respect, many studies have explored the use of organic fertilizers to reduce chemical P fertilizer application and enhance P fertilizer use efficiency (Hua and Zhu, 2020; Zhang et al., 2021d). It has also been shown that various types of organic materials can significantly reduce soil total P leaching losses (Zhang et al., 2021c), underscoring the pivotal role of organic materials in meeting the P demand for vegetable production.

Over the years, numerous studies have demonstrated that the application of organic materials to agricultural land affects soil P biogeochemical processes by altering P forms. It is now understood that unstable P pools are more susceptible to changes due to fertilization (Zhang et al., 2021c). The application of organic materials has been shown to promote the conversion of soil P into labile P or microbial biomass phosphorus (MBP), thereby reducing the deposition of stable P and improving soil P bioavailability (Liu et al., 2020a; Fei et al., 2022). For instance, cattle manure, as a P-efficient organic material, can enhance soil microbial diversity, improve soil aeration and water-holding capacity, and promote pepper yield (Xiang et al., 2022; Hernandez et al., 2023; Wang et al., 2023b). However, the impact of the optimal manure substitution rate on soil P transformation and availability remains uncertain, especially in a red soil with high P fixation capacity.

Crop production, which relies on manure application, responds to root traits and the rhizosphere. An increasing body of evidence suggests that soil organic phosphorus (P_o_) cycling is highly dependent on the interaction between roots and the rhizosphere (Amadou et al., 2021; Zhang et al., 2023a). Long-term field trials have shown that a combination of organic and inorganic applications can synergistically improve root morphology and enhance the rhizosphere soil environment, including soil enzyme activity and root secretion content, effectively increasing crop yields (Zhang et al., 2023b). There is a rich literature available substantiating that increased organic fertilizer application leads to changes in the rhizosphere microbiome community structure, increased abundance and diversity of bacteria carrying *phoD* functional genes, and enhanced soil phosphatase activity, which, in turn, drives P_o_ mineralization (Lu et al., 2023a). It has also been suggested that organic fertilizer enhance P effectiveness by inducing changes in soil pH and increasing soil organic carbon (SOC) levels, which release adsorbed P (Nobile et al., 2020). Additionally, the crop root system can improve its capacity to access P through its physiological functions, such as increasing root dry weight, root density, extending fine roots, root hairs, and establishing P uptake channels through symbiosis with AM fungi (Wang and Lambers, 2020; Amadou et al., 2021; Liu, 2021; Wang et al., 2022). However, little is currently known about how root and rhizosphere processes correspond to soil P transformations at varying P replacement levels.

This study aims to test the following hypotheses: (1) Soil labile P pools gradually decrease with increasing levels of manure as a substitute for P fertilizer. (2) The development of the pepper root system initially increases and then decreases with higher levels of manure substitution for P fertilizer. The rhizosphere soil environment improves with manure application and stabilizes with increasing manure application. (3) The interconversion between soil P pools plays a crucial role in driving the crop root system to forage for P. Importantly, we sought to determine the optimal matching ratio that balances crop yield and farmland environment sustainability in vegetable systems and quantify the contributions of soil P transformation and crop root and rhizosphere processes to efficient crop P uptake, elucidating the root-rhizosphere-soil interaction mechanisms underlying manure substitution for chemical P fertilizer.

## 2 Materials and methods

### 2.1 Soil and materials

A pot experiment was established in the solar greenhouse at the National Purple Soil Monitoring Station in Beibei, Chongqing, China (29°48’ N, 106°24’ E) during the 2022 growing season (March-June). The selected pepper variety was “Xinxiang No. 8”, a hybrid variety from Jiangxi Nongwang High-tech Limited Company. Cow manure was sourced from Chongqing Zheshu Agricultural Science and Technology Development Limited Company. The basic soil properties were as follows: pH 5.9, soil organic matter 34.7 g·kg^-1^, total nitrogen 0.71 g·kg^-1^, Olsen-P 36.1 mg·kg^-1^, alkaline dissolved nitrogen 132.3 mg·kg^-1^, and available potassium 65.7 mg·kg^-1^. The soil was collected from Shilin County, Yunnan Province, characterized as ultisol (US Soil Taxonomy), with a clay texture (Wang et al., 2023b). The basic soil properties were as follows: pH 5.9, soil organic matter 34.7 g·kg^-1^, total nitrogen 0.71 g·kg^-1^, Olsen-P 36.1 mg·kg^-1^, alkaline dissolved nitrogen 132.3 mg·kg^-1^, and available potassium 5.7 mg·kg^-1^.

### 2.2 Experimental design

The experiment comprised nine treatments with pots of 16 cm in diameter and 18 cm in height (**Supplementary Figure S1**), including a control with no P fertilizer (CK) and eight P fertilizer substitution levels using manure based on the total P content: fully chemical fertilizer (0M), 10% (10M), 20% (20M), 30% (30M), 40% (40M), 50% (50M), 75% (75M), and 100% (100M). Each treatment was replicated four times. Each pot contained 4.0 kg of dry soil, and three uniform seedlings were transplanted into each pot. All treatments received N (200 mg·kg^-1^ dry soil), P_2_O_5_ (550 mg·kg^-1^ dry soil), and K_2_O (480 mg·kg^-1^ dry soil) except for the no P control. The manure application rate was determined based on the total P substitution rate, and any remaining nutrient deficiencies were supplemented with chemical fertilizers (urea, calcium superphosphate, potassium chloride) to achieve the same level. The soil and fertilizer were thoroughly mixed before transplanting. Pepper seeds were disinfected with carbendazim, subsequently wrapped in nutrient-rich paper, and placed in a nutrient solution for seedling cultivation (Liu et al., 2017). After 40 days, uniformly grown pepper seedlings were selected for transplanting and watering, with soil water content maintained at approximately 70% of the field water holding capacity during the experimental period.

### 2.3 Sample collection and analysis

Samples were collected after 60 days (flowering stage) of potting culture for the following analysis: Plant parts (aboveground and roots) were washed, dried separately with deionized water, and heated at 105°C for 30 minutes to terminate enzymatic activity. Subsequently, they were dried to a constant weight at 65°C, milled using a ball milling machine, and stored for measurement. Soil samples were divided into two portions, with one portion dried in its natural state and sieved through 2 mm and 0.15 mm sieves for the analysis of soil physicochemical properties. The other portion was stored in a refrigerator at -20°C for analysis of soil MBP.

This study conducted the analysis of each indicator following standard soil agrochemical analysis methods. Plants were digested by the HNO_3_-H_2_O_2_ method and analyzed for nutrients (ICP-OES). Root morphology was scanned using a root scanner (Epson Perfection V850 Pro) and imported the data into Win-RHIZO software (Win-RHIZO Pro 2004b, Version 5.0, Canada) for scanning and digitization. Arbuscular mycorrhizal colonization was determined through microscopic observation after staining with Trypan Blue Stain Solution (Giovannetti and Mosse, 1980). The determination of soil properties involved various methods: SOC was determined by the potassium dichromate volumetric method (Bao, 2000); Olsen-P and CaCl_2_-P were extracted by 0.5 mol·L^-1^ NaHCO_3_ (pH 8.5, soil-water ratio 1:20) and 0.01 mol·L^-1^ CaCl_2_ (soil-water ratio 1:5) respectively and determined by the molybdovanado phosphatase method (Zhang et al., 2021b). Soil pH was determined by the potentiometric method (1:2.5 *w*/*v* in water) (FiveEasy Plus FE28). Soil acid phosphatase (ACP) and alkaline phosphatase (ALP) were determined using the colorimetric method of disodium benzene phosphate. Phytase (S-Phytase) was determined spectrophotometrically. *β*-glucosidase (S-*β*-GC) using 3,5-dinitrosalicylic acid (DNS) colorimetric method. MBP was analyzed by chloroform fumigation (Brookes et al., 1982). To categorize soil P forms, we followed the sequential extraction method proposed by Tiessen and Moir (1993), which divided P into nine types (Hedley et al., 1982), including Resin-P, NaHCO_3_-P, NaOH-P, HCl-P, and Residual-P. Notably, NaHCO_3_-P, NaOH-P, and HCl-P encompassed both organic and inorganic fractions. Based on the bioavailability of various P fractions, they are classified into four distinct P pools: the labile P pool consists of Resin-P, NaHCO_3_-P_i_ and NaHCO_3_-P_o_; the moderately labile P pool includes NaOH-P_i_, NaOH-P_o_ and dil.HCl-P_i_; the sparingly labile P pool is made up of conc.HCl-P_i_ and conc.HCl-P_o_; and the Residual-P is considered to be within the non-labile P pool.

Total RNA from pepper root was extracted using the EZ-10 DNAaway RNA extraction kit. RNA integrity and concentration were determined by the NanoPhotometer Analyzer (N50 Touch, Serial No.: T51314) and 1% agarose gel, respectively. The cDNA was synthesized by reverse transcription using PrimeScript TM RT reagent kit with gDNA Eraser (Takara) and subjected to qPCR (StepOnePlus Real-Time PCR System, Applied Biosystems, Q1), Reaction conditions were: 95°C for 10.0 min, followed by 40 cycles of 95°C for 15.0 s, 60°C for 1.0 min. The relative expression levels of the target genes were calculated using the 2^−ΔΔCt^ method relative to reference genes (Livak and Schmittgen, 2001). All the primers used are provided in **Supplementary Table S1**.

### 2.4 Data analysis and statistics

The following formula was used to calculate plant P accumulation (Jamal et al., 2023).


Plant P accumulation=Aboveground biomass∗Aboveground P concentration+Root biomass∗Root P concentration

All data were analyzed by one-way ANOVA using SPSS 26. In cases where the ANOVA indicated significance, we compared means using the LSD test at a significance level of *P* < 0.05. All figures in this study were generated using Origin 2023.

## 3 Results

### 3.1 Pepper biomass, P concentration, and accumulation response to manure substitution

**Figure 1:**
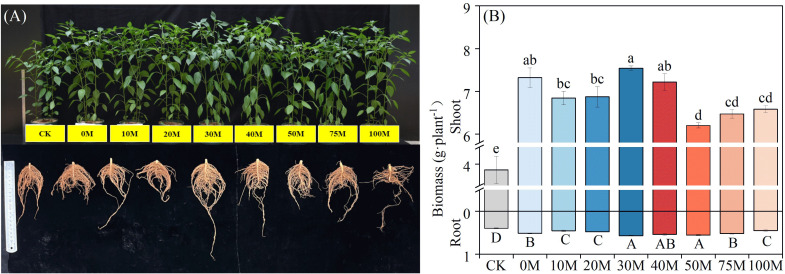
Pepper phenotype **(A)** and biomass **(B)** of shoot and root under different manure substitution rates. Different lowercase and uppercase letters indicate significant differences between the aboveground and root of different treatments, respectively (*P* < 0.05).

The aboveground and root biomass of pepper exhibited significant increases under all P treatments compared to the control, with a consistent trend in both plant parts (*P* < 0.05) (**Figure 1**). Aboveground biomass showed a substantial increase, ranging from 60.6% to 95.3% in the P application treatments compared to the no P control. Aboveground biomass was similar to the 0M treatment for manure substitution rates below 40%. The peak biomass occurred at the 30% rate, registering 1.15 times higher than that of the 100% manure treatment. However, the aboveground biomass was significantly suppressed at substitution rates exceeding 50%, reaching only 84.7% to 89.9% of the biomass observed with the chemical fertilizer treatment. In contrast, root growth responded differently. Root biomass was promoted to varying degrees at different substitution rates compared to the control, increasing by approximately 14.3% to 42.9%. When compared to the chemical fertilizer treatment, both low substitution rates (≤20%) and the full manure treatment significantly inhibited pepper root growth. Root biomass levels were similar at substitution rates between 30% and 50%, increasing by 4.4% to 11.1% and 17.5% to 25.0% when compared to the 0M and 100M treatments, respectively.

**Figure 2:**
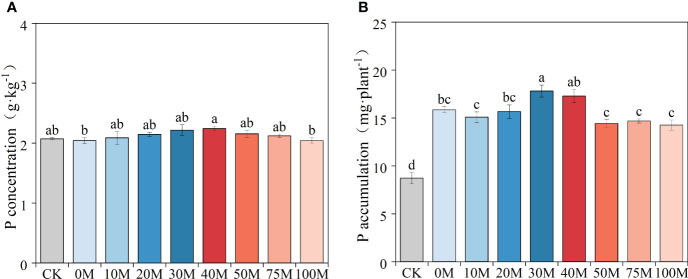
Total P concentration **(A)** and accumulation **(B)** under different manure substitution rates. Different lowercase letters indicate significant differences among the different treatments (*P* < 0.05).

Total P concentration in pepper exhibited an overall increasing and then decreasing trend at different substitution rates (*P* < 0.05) (**Figure 2A**). The highest concentration was observed under the 40M treatment, which was 9.8% higher than both fully chemical and fully organic fertilizer treatments. Total P accumulation in pepper followed a similar trend as total biomass, with total P uptake under P application treatments being 63.6% to 104.4% higher than the control. The highest pepper P uptake was observed at manure substitution rates of 30% to 40%, significantly exceeding the chemical fertilizer treatment and high substitution rate treatments (50M, 75M, and 100M) (**Figure 2B**).

### 3.2 Changes in root/rhizosphere traits response to manure substitution rate

**Table 1** summarizes the differences in pepper root traits under various manure substitution levels. The root length, root surface area, and root diameter under CK treatment were 68.2% to 91.8%, 71.4% to 86.1%, and 63.0% to 96.7% of the P treatment, respectively. Root length and root diameter exhibited an increasing and then decreasing trend with increasing substitution rates, with peak values observed at 30% to 40% substitution rates. Total root length did not differ significantly between the 0M treatment and low substitution rate treatments but was suppressed when the rate exceeded 50%. For example, the 100M treatment displayed only 77.5% and 74.3% of the root length compared to the 0M and 30M treatments, respectively. The application of chemical fertilizer alone significantly reduced root diameter by approximately 32.6% compared to the 40M treatment.

**Table 1:** Root length, root surface area, root diameter, and arbuscular mycorrhiza colonization of pepper under different manure substitution rates.

Treatment	Root length (m·plant^-1^)	Root surface area (cm^2^·plant^-1^)	Root diameter (mm)	AM colonization (%)
CK	9.8 c	96.2 b	0.29 b	11.5 bc
0M	13.8 a	123.5 ab	0.31 b	2.0 e
10M	12.7 ab	113.2 ab	0.30 b	4.0 de
20M	13.8 a	111.7 ab	0.31 b	6.3 d
30M	14.4 a	134.7 a	0.41 ab	9.3 c
40M	14.0 a	120.1 ab	0.46 a	10.0 c
50M	12.0 abc	123.7 ab	0.36 ab	11.5 bc
75M	12.3 abc	123.2 ab	0.38 ab	14.3 a
100M	10.7 bc	115.3 ab	0.37 ab	12.5 ab

In each column, means followed by a common letter are not significantly different at the 5% probability level according to the LSD test.

The AM colonization was significantly reduced by 82.6% under the 0M treatment compared to the control. Overall, the colonization rate initially increased and then decreased with an increased manure substitution rate. The maximum colonization was observed under the 75M treatment, which increased by 615.0% and 14.4% compared to the 0M and 100M treatments, respectively, with no significant difference between 100M and 75M.

**Figure 3:**
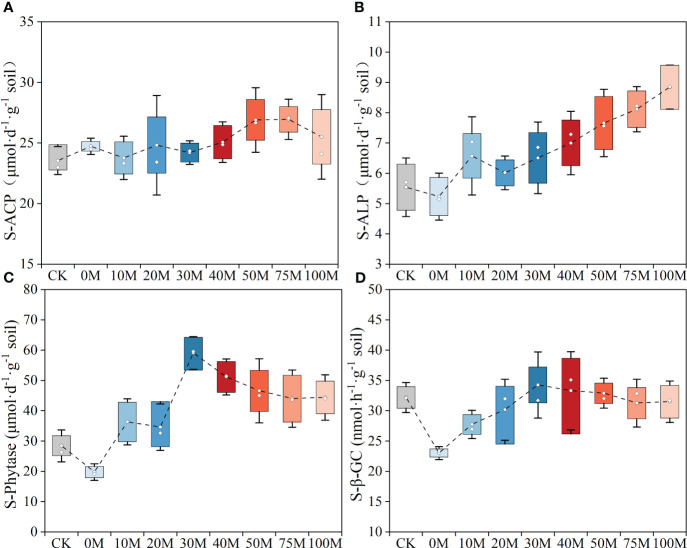
Changes in soil acid phosphatase **(A)**, alkaline phosphatase **(B)**, phytase **(C)**, and *β*-glucosidase **(D)** activities under different manure substitution rates. The hollow circle on the box represents the average number of treatments, and the hollow diamond represents the median of each treatment.

Soil nutrient cycling enzymes exhibited distinct responses to each manure substitution level (**Figure 3**). Acid phosphatase activity remained unaffected by P fertilizer application and manure substitution rate, while alkaline phosphatase activity gradually increased with increasing substitution rate. Compared to CK, the chemical fertilizer treatment inhibited alkaline phosphatase activity, whereas the manure substitution treatment yielded positive effects. Alkaline phosphatase activity was 1.34 to 1.61-fold higher than the 0M treatment when the substitution rate was 40% or higher. Similarly, phytase activity exhibited inhibition under the 0M treatment. However, phytase activity increased initially and then decreased with the increasing manure application rate, with the highest activity recorded at the 30% substitution rate, showing increases of 125.6% and 200.0% compared to the 0M and 100M treatments, respectively. Soil *β*-glucosidase activity increased and then stabilized with the increasing substitution rate. Soil *β*-glucosidase activity under 0M treatment was 21.6% lower than the control. The highest enzyme activity was observed with 30% substitution rate, exhibiting an increase of 16.8% compared to the CK and 48.9% compared to the 0M treatments. In addition, the relative expression of phosphate transporter protein genes (*PHT1.1*, *PHT1.2*, and *PHT1.6*) was upregulated in pepper roots under manure treatments compared to the 0M treatment, with the highest expression levels observed in the 30M treatment. Excessive substitution rates led to decreased expression levels compared to the 30M treatment (**Supplementary Figure S2**).

### 3.3 Soil available P and P fractions affected by manure substitution

Soil Olsen-P and CaCl_2_-P exhibited consistent overall trends (**Table 2**). P content was significantly higher in all P fertilizer treatments compared to the no P control and decreased gradually with increasing manure substitution rate. Soil Olsen-P content under the 0M treatment was significantly higher than that under the 30% to 100% substitution rate treatments, with increases of 9.4%, 11.4%, 13.5%, 18.5%, and 32.7%, respectively, while it did not differ from the ≤20% substitution rate treatments. CaCl_2_-P content was highest under the 0M treatment at 1.13 mg·kg^-1^, which was 4.7 folds higher than the no P control. Compared to the 0M treatment, CaCl_2_-P levels were comparable when the substitution rate was ≤30%, but they decreased significantly for substitution rates of 40% to 100%, with decreases of 21.2%, 36.3%, 54.9%, and 57.5%, respectively. P fertilizer also yielded a significant enhancement effect on MBP, with soil MBP levels under P fertilizer treatments being 2.2 to 3.2 folds higher than the control. There was no difference in MBP levels under the 0M and 30M treatments, which were significantly higher than the 100M treatment. Furthermore, SOM content increased progressively with increasing substitution rate, with increases of 10.0%, 10.6%, 12.8%, and 12.7% observed when the rate was 40% or higher compared to the 0M treatment. However, there were no difference among all manure treatments for SOM content. Soil pH exhibited significant differences under all treatments. Compared to CK, the application of P fertilizer significantly increased soil pH, with the pH gradually increasing with the manure substitution rate.

**Table 2:** Changes in soil Olsen-P, CaCl_2_-P, SOM, pH, and MBP under different manure substitution rates.

Treatment	Olsen-P (mg·kg^-1^)	CaCl_2_-P (mg·kg^-1^)	SOM (g·kg^-1^)	pH	MBP (mg·kg^-1^)
CK	20.5 e	0.24 e	34.6 b	5.66 i	111 c
0M	51.2 a	1.13 a	34.8 b	5.75 h	360 a
10M	51.7 a	1.02 ab	36.4 ab	5.87 g	/
20M	50.2 a	0.98 ab	36.6 ab	6.12 f	/
30M	46.8 b	0.93 ab	36.6 ab	6.34 e	343 a
40M	46.0 b	0.89 bc	38.3 a	6.55 d	/
50M	45.1 bc	0.72 c	38.5 a	6.72 c	/
75M	43.2 c	0.51 d	39.2 a	6.93 b	/
100M	38.6 d	0.48 d	39.2 a	7.03 a	247 b

In each column, means followed by a common letter are not significantly different at the 5% probability level according to the LSD test. “/” indicates no data.

**Figure 4:**
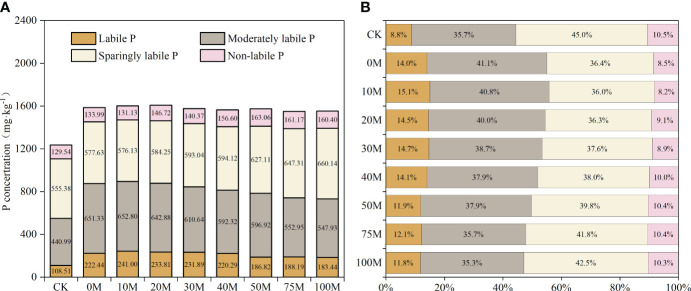
Changes in the content **(A)** and percentage **(B)** of four soil P pools at different manure substitution rates.

The levels of the nine soil P fractions, based on sequential extraction, differed in response to each substitution rate. Various P forms were categorized into four P pools: labile P, moderately labile P, sparingly labile P, and non-labile P. The P content and percentage of these P pools are shown in **Figure 4**. All P application treatments increased the content of each soil P pool compared to CK, with the greatest effect on the enhancement of labile P (increasing by 3.0% to 6.3%), while sparingly labile P decreased by 2.5% to 9.0%. Soil labile P and moderately labile P content and percentage decreased gradually with increasing substitution rate, while sparingly labile P and non-labile P exhibited the opposite trend. Soil P forms were primarily dominated by moderately labile P and sparingly labile P, accounting for about 75.9% to 80.7% of the total percentage. There was no reduction in the percentage of the soil labile P pool when the substitution rate was 40% or lower compared to the 0M treatment.

### 3.4 Relationship between soil properties and P fractions

**Figure 5:**
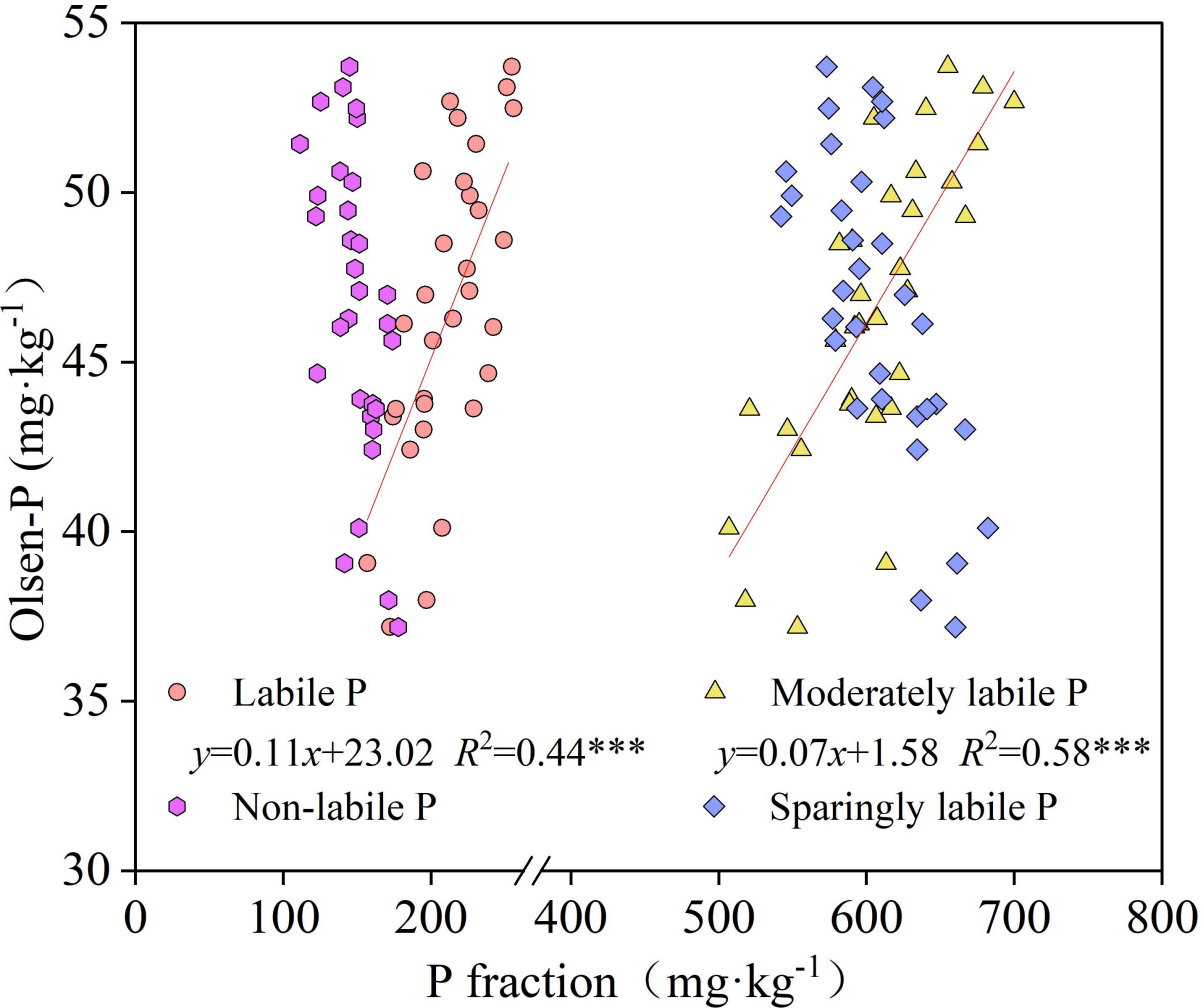
Relationship between soil Olsen-P and the four P pools (*** indicates significant regression at *P* < 0.001).

As shown in **Figure 5**, both soil labile P (*R* ^2^ = 0.44) and moderately labile P (*R* ^2^ = 0.58) were significantly positively correlated with Olsen-P when fitting soil P pools to available P correlations (*P* < 0.001), indicating that soil Olsen-P increased with increasing levels of these two P pools. Among the nine P forms, NaOH-P_o_ showed the highest correlation with Olsen-P, with an *R* ^2^ value of 0.60 (**Supplementary Figure S3**).

**Figure 6:**
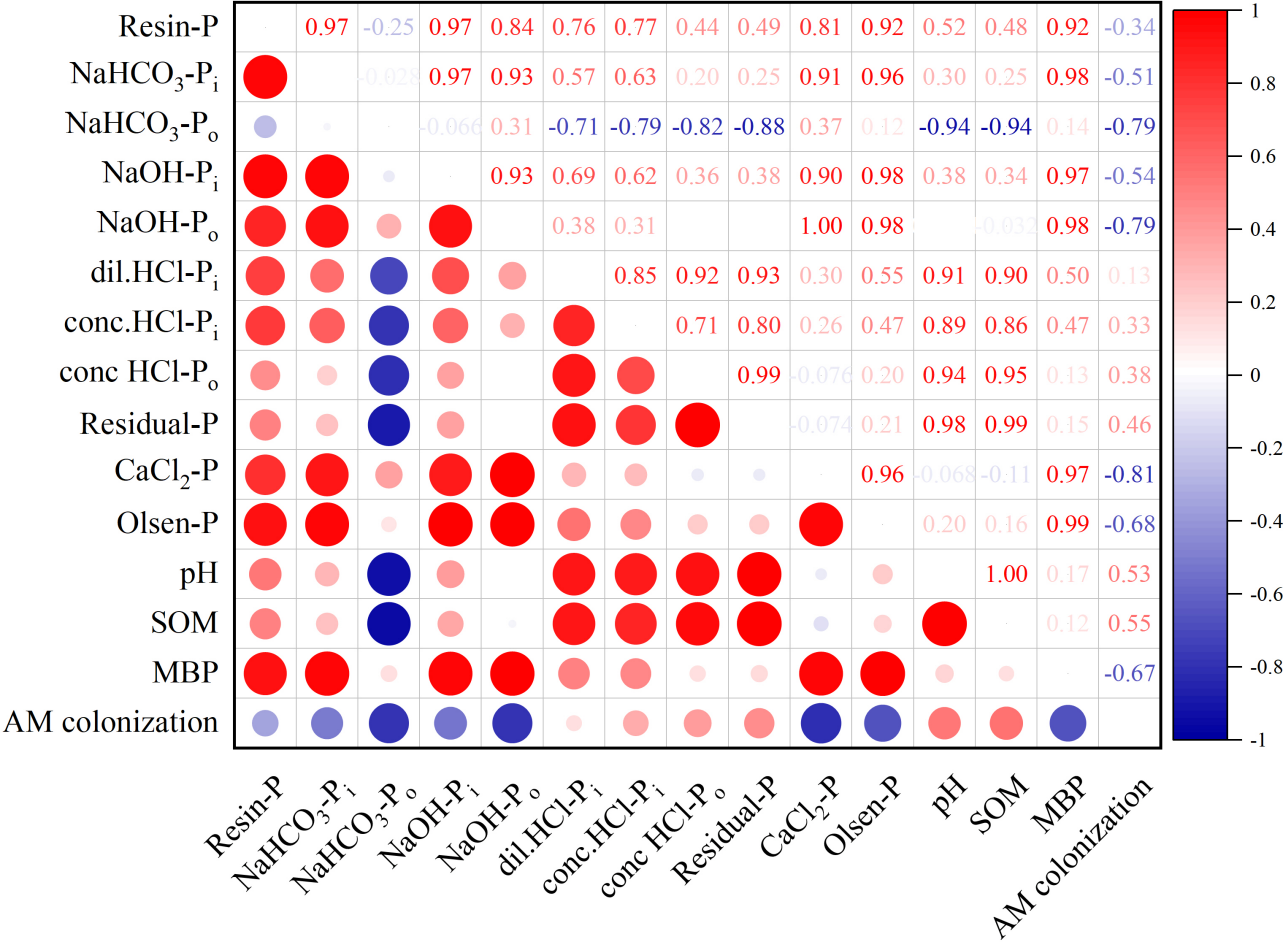
Correlation analysis of four P pools with soil properties (red and blue indicate positive and negative correlations, respectively; the shade of color and the size of the circle indicate the size of the correlation).

The correlation heat map in **Figure 6** revealed that soil Olsen-P and CaCl_2_-P were primarily influenced by Resin-P, NaHCO_3_-P_i_, NaOH-P_i_, and NaOH-P_o_. These four P forms were significantly positively correlated with each other, suggesting their susceptibility to morphological transformations. NaHCO_3_-P_o_ exhibited a significant negative correlation with some constituents of the sparingly labile P and non-labile P, indicating that NaHCO_3_-P_o_ is predominantly converted to a highly effective P source for crop uptake after mineralization. Conc.HCl-P_i_ and conc.HCl-P_o_, as constituents of the sparingly labile P pool, exhibited an overall positive correlation with other P forms, with the order of positive correlation being Residual-P > dil.HCl-P_i_ > Resin-P > NaOH-P_i_ > NaHCO_3_-P_i_. This suggested that conc.HCl-P_i_ and conc.HCl-P_o_ were more readily immobilized or converted to partially moderately labile P forms. Soil MBP was significantly positively correlated with Olsen-P, while AM colonization showed a negative correlation, indicating a significant antagonistic relationship between P transformation and AM colonization.

## 4 Discussion

### 4.1 Effect of manure substitution for chemical P fertilizer on biomass and P absorption of pepper

The substitution rate of manure for chemical fertilizers is a key factor in determining crop yield. In this study, pepper biomass and P uptake with 30%–40% manure substitution were equal to or higher than the chemical P fertilizer treatment (**Figures 1**, **2**). This enhancement was credited to the integrative impact of the fertilization regime on exploiting the root biological potential and advancing soil P turnover. However, exceeding 50% of the total P with manure P significantly reduced both crop biomass and P uptake. This decline was ostensibly linked to a deficiency of labile P concentration, which proved inadequate for sustaining essential root development and nutrient uptake. Previous research on substituting organic fertilizer for nitrogen fertilizer has shown that moderate substitution levels (35%–70%) yield the greatest impact on vegetable yields, while fruiting vegetables benefit from lower substitution rates (<35%) (Li et al., 2021b; Wang et al., 2023a). While, maize crops have shown good growth and development under high manure inputs, improving nutrient use efficiency (Otinga et al., 2013). This variation is attributed to the characteristics of vegetables, such as their shallower root systems and higher nutrient demand, which make them more responsive to external nutrient inputs (Zhang et al., 2010). Current evidence suggests that the uptake of P by crops is mainly in the form of phosphate and is limited by short-term demand, reducing the role of a single P source, whether it is inorganic or organic phosphate. Previous study notes that simply converting to 100% organic production will inevitably lead to increased agricultural land use (Muller et al., 2017), and it can be challenging to maintain crop production even when organic P fertilizer is applied exclusively to cereal crops with well-developed root systems (Andriamananjara et al., 2019). Replacing 40% of P fertilizer with organic P fertilizer has been found to be more effective in improving the soil environment and crop production (Chen et al., 2021). The application of a manure substitution strategy on P can ensure crop production while promoting resource recycling and integrating agriculture and livestock. However, little is currently known on manure substitution based on P fertilizers, nor is it clear whether the manure substitution rate varies under different soil P level environments. Therefore, more research should be conducted to inform regional production practices in future farmland management.

### 4.2 Root/rhizosphere response under manure substitution strategy

The root-rhizosphere interface represents the most frequent and sensitive area for material exchange between plants and soil, making it essential to understand crop root systems and rhizosphere processes when evaluating fertilization practices (Zhang et al., 2023a). In this study, manure P substitution at 30%–40% of chemical P yielded the most significant positive impact on root traits, and AM colonization increased with higher manure application rates. This phenomenon can be attributed to the regulation of AM colonization by various factors. The application of organic fertilizers has been shown to affect crop nutrient acquisition through the AM pathway, but the effect, whether positive or negative, remains inconclusive (Fornara et al., 2020; Lu et al., 2023b). Furthermore, AM fungi can use carbon sources from organic fertilizers to increase their biomass, and the colonization rate is partly regulated by soil P levels (Jiang et al., 2021). The present study found that AM colonization was regulated by the ratio of organic and inorganic P fertilizers. Excessively low or high manure substitution rates were inhibited by high P levels and carbon oversaturation, respectively, while moderate available P levels and organic carbon inputs created a favorable soil environment for AM colonization and sporulation, in line with the literature (Zhang et al., 2021a).

The application of a combination of organic and inorganic fertilizers is an effective approach to increase soil enzyme activity and accelerate P turnover (Zhang et al., 2022). In the current study, alkaline phosphatase activity increased gradually with higher manure substitution rates, while acid phosphatase was not affected by the fertilization strategy, consistent with previous research (Spohn and Kuzyakov, 2013). Research has shown that acid phosphatase is mainly secreted by living plants, while alkaline phosphatase is significantly influenced by soil microorganisms (Krämer and Green, 2000; Wan et al., 2021). Therefore, under manure substitution measures, the dynamics of soil enzymatic activity may be influenced by changes in soil microbial communities. Notably, phytase activity was suppressed under the fully chemical fertilizer strategy, but it showed an increasing and then decreasing trend with organic management. Another study observed that phytase activity increased to a certain level and then stabilized as manure substitution rates increased (Li et al., 2012). This phenomenon is related to the biological fixation of P by soil microorganisms, with the fixed P primarily consisting of P_o_, such as DNA and phospholipids (Peng et al., 2021), which is converted into plant-available forms through phytase digestion (Azeem et al., 2015).

### 4.3 P effectiveness and characterization of P forms under manure substitution strategy

Olsen-P is often used to characterize soil P that is readily available for plant uptake, while CaCl_2_-P indicates the fraction dissolved in the soil solution (Zhang et al., 2021b). In this study, the 0M and low substitution (≤20%) treatments exhibited similar soil Olsen-P levels. Chemical P fertilizers play a crucial role in increasing soil P effectiveness; however, manure P is released too slowly to meet crop demand. This characteristic of chemical P fertilizers resulted in significantly higher soil CaCl_2_-P levels compared to other treatments when the substitution rate was ≤30%, increasing soil environmental stress. It is well-established that soil MBP is biologically fixed by soil microorganisms and exhibits an extremely fast turnover rate (Peng et al., 2022). MBP serves as a supplementary energy source for plant-available P and plays a role as a “transit station” in the soil P cycle (Zhang et al., 2018). In the present study, MBP content was similar to the fully chemical P treatment when the substitution rate was 30% and could maintain a high potential for P supply, but it significantly decreased under the 100M treatment. Therefore, substituting 30%–40% of P fertilizer with manure is a green and sustainable management strategy that balances crop production and soil health. Previous studies have suggested that partial organic fertilizer treatments tend to result in higher available P levels, which differs from the results of this study. The monitoring of soil runoff indicated that soil available P loss under chemical fertilizer treatment was much higher than organic fertilizer treatment, and soil P loss and degree of P saturation (DPS) decreased significantly with 30% manure substitution, reducing the risk of P loss (Li et al., 2021a; Jin et al., 2023). However, this study was conducted in a closed environment with minimal P loss. In a long-term field study, soil available P under 100% manure treatment was much lower than under chemical fertilizer treatment, resulting in lower yields (Lu et al., 2021), consistent with the results of this study.

Soil P fractionation provides insights into the soil P supply capacity to crops. More active P components are preferable for direct crop uptake and utilization. The present study found that the percentage of soil labile P was higher when the manure substitution rate was less than 40% compared to the chemical fertilizer treatment. In other words, manure substitution of less than 40% ensures that nutrient requirements for crops are met in a timely manner. Moderately labile P gradually decreased with increasing substitution rate, while sparingly labile P and non-labile P exhibited the opposite trend, which matched soil Olsen-P. Additionally, the transformation of soil P pools is regulated by soil environmental factors, including soil pH and SOM. The correlation heatmap reveals that soil environmental factors are significantly correlated with various P components. Soil P effectiveness is an important factor in crop yield response to P fertilization and is often used as a guide for agricultural production (Buczko et al., 2018; Meyer et al., 2020; Ibrahim et al., 2022). Many studies have shown that P fertilizers with organic materials increase crop productivity by enhancing plant-available P (Carneiro et al., 2021; Jamal et al., 2023). In this study, excessively high manure application rates led to a significant reduction in soil P effectiveness, which was the primary reason for the reduced growth of pepper.

### 4.4 Efficient application of manure based on P management

At present, there is a considerable amount of organic resources in livestock development, but the recovery rate is less than 50% (Liu, 2018; Liu et al., 2023). Additionally, P reserves in China are depleting (Liu et al., 2020b). Indeed, returning organic resources to farmland is an effective solution to address these challenges and achieve a balance between crop yield and environmental sustainability in agriculture. Partial substitution strategies for manure based on P can maximize crop biological potential (root traits, AM colonization, etc.), enhance soil fertility, promote soil P turnover, and reduce the risk of nutrient surpluses. Overall, for vegetable crops with underdeveloped root systems, especially pepper, regulating the manure substitution rate at 30%–40% offers the most advantages. While 100% manure models improve the soil environment, they may lead to reduced crop productivity. This is also influenced by the duration of organic fertilizer management practices. Current evidence has demonstrated that under a regime of sustained manure substitution, the discontinuation of fertilization does not precipitate a decline in soil available P levels, which remain comparatively high for at least two years, or possibly even longer (De Figueiredo et al., 2020). In other words, under the extended organic management, it may be necessary to further reduce the optimal rate of manure substitution, and to implement controls on the total P application in order to mitigate environmental risks.

## 5 Conclusion

The manure substitution rate is a determining factor in crop productivity and soil P turnover under equal P input conditions. Crops can enhance root trait development, increase biomass and P uptake, and maintain moderate soil available P and MBP levels with manure substitution of 30%–40% for chemical P. Both excessively high (≥50% manure P) and low (≤20% manure P) manure substitution rates can reduce pepper productivity. Additionally, the analysis of soil P sequential fractionation showed that when the substitution rate was below 40%, the percentage of labile P in the soil remained stable at 14.1%–15.1%. These levels were found to be comparable to those observed in the complete chemical fertilizer treatment. Therefore, substituting 30%–40% of chemical P with manure ensures crop production and soil health and has practical significance for guiding environmentally friendly vegetable production, especially for crops like pepper. The efficient transformation of P in organic fertilizers can also be influenced by factors such as the source of organic fertilizer (ruminant and monogastric animals), composting processes, and microbial action. Therefore, further research is still needed in the future on organic fertilizer application methods based on different organic fertilizer sources and environmental conditions to attain green agricultural development.

## Data availability statement

The original contributions presented in the study are included in the article/**Supplementary Material**. Further inquiries can be directed to the corresponding author.

## Author contributions

KS: Conceptualization, Data curation, Formal analysis, Investigation, Visualization, Writing – original draft, Writing – review & editing. YC: Investigation, Writing – review & editing. LS: Investigation, Writing – review & editing. BW: Investigation, Writing – review & editing. YW: Writing – review & editing. SL: Investigation, Writing – review & editing. CZ: Investigation, Writing – review & editing. YW: Conceptualization, Writing – review & editing. WZ: Conceptualization, Formal analysis, Funding acquisition, Writing – review & editing.
