# Supplementary material for: Optimizing the manure substitution rate based on phosphorus fertilizer to enhance soil phosphorus turnover and root uptake in pepper (Capsicum)
Source: Front Plant Sci. 2024 Mar 5;15:1356861. doi: 10.3389/fpls.2024.1356861 (PMC10948398; doi:10.3389/fpls.2024.1356861)
Supplement: Supplementary file 1 [file DataSheet_1.docx]

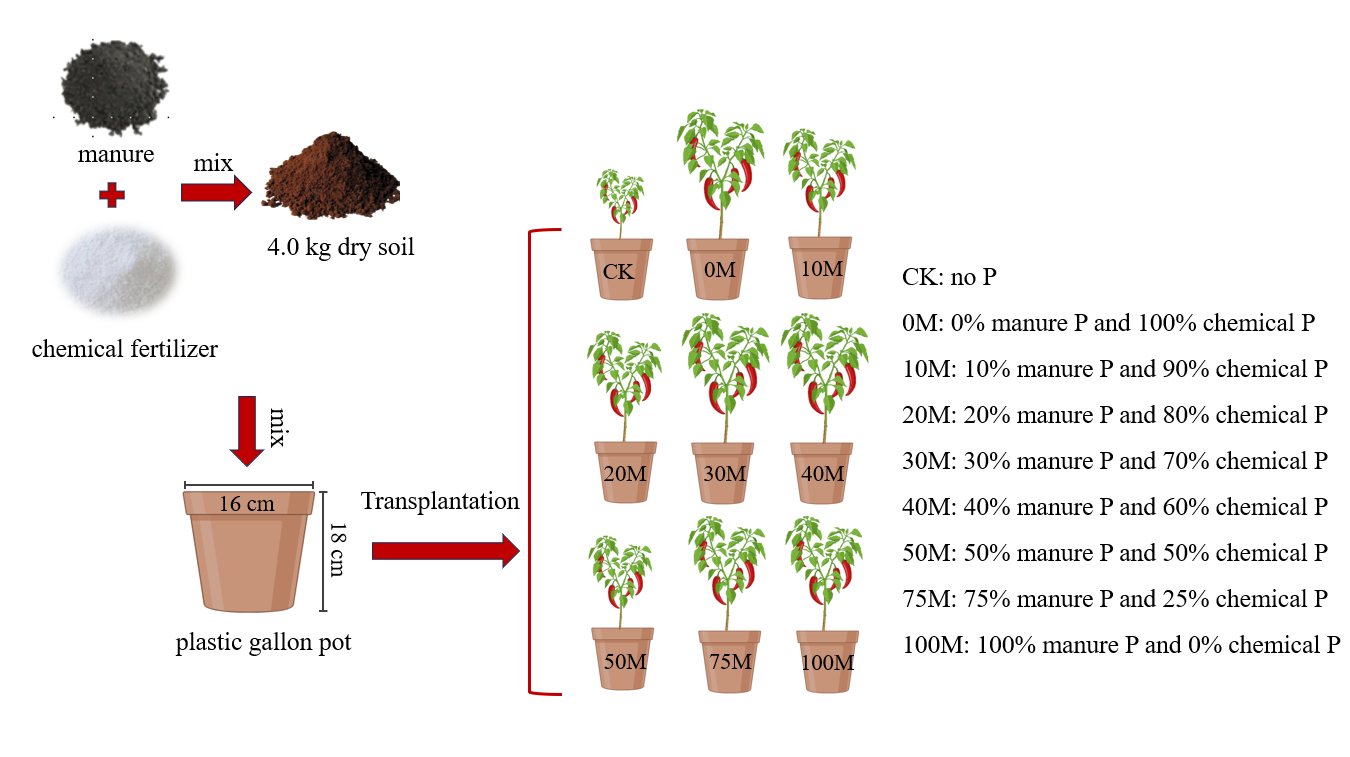


**Fig. S1** Experimental procedure and material information.
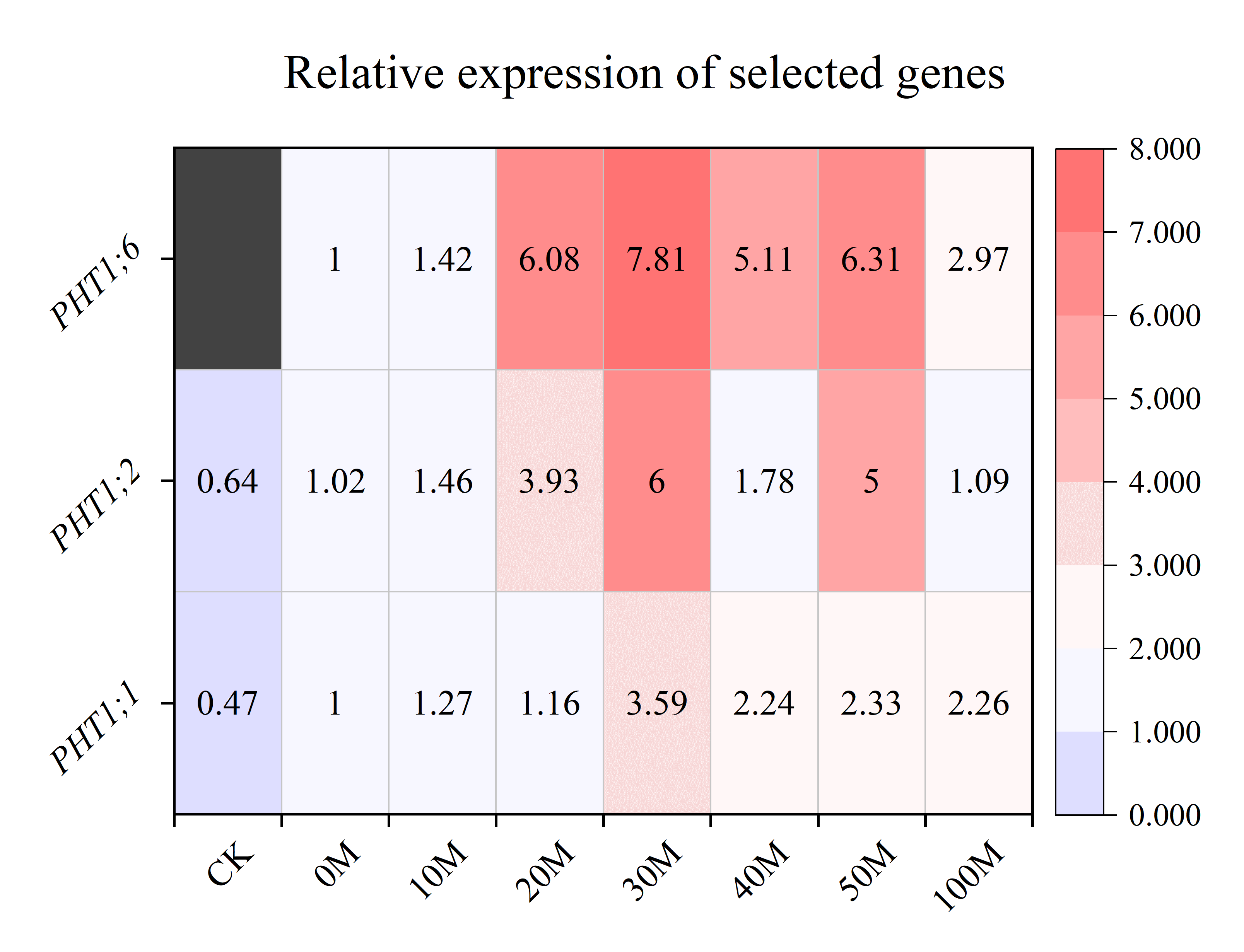


**Fig. S2** Relative expression of selected genes under different manure substitution rates in pepper

**
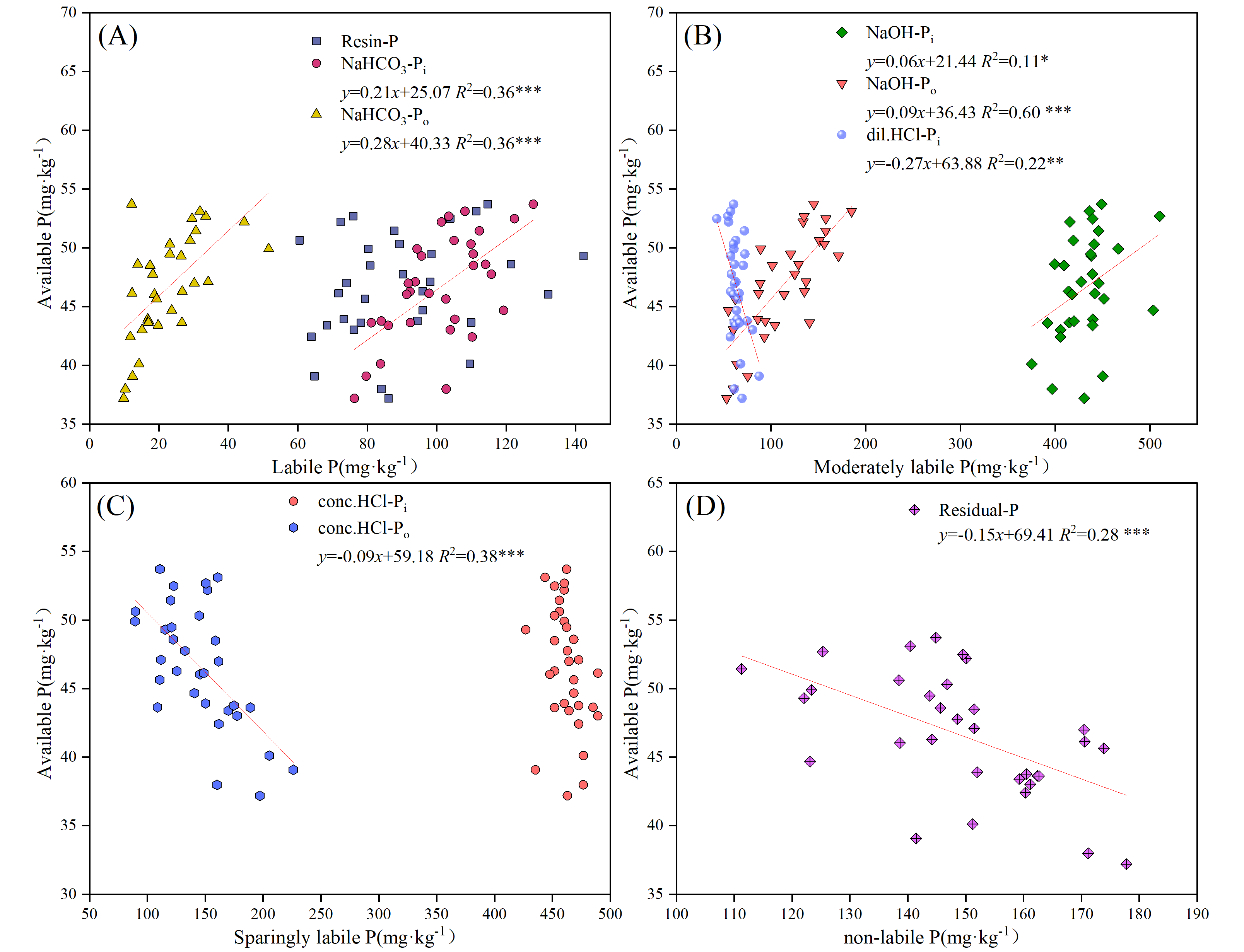
Fig. S3** Relationships between available P and soil labile P (A), moderately labile P (B), sparingly labile P (C), and non-labile P (D)

**Table S1:** Gene-specific primer sequences of *C. annuum* phosphate transporter genes used in qRT-PCR expression analysis.

| **S. No.** | **Locus ID** | **Forward primer** | **Reverse primer** |
| --- | --- | --- | --- |
| 1 | *LOC107864586\|PHT1;1* | GCAATTGTGATTGCTGGCATGGGC | GGGTGCCACAGAAGGCGACTCCG |
| 2 | *LOC107866602\|PHT1;2* | TGGTGGAATTGTCGCACTTA | AGCTTCAGGAGGGGTTGAAT |
| 3 | *LOC107875070\|PHT1;6* | GCACTTGACACAGCACGTACCC | CAAGTTTATCTCCCAGCCAACC |
|  | *ACTIN* | TTCCGTTGCCCAGAGGTCCT | GGGAGCCAAGGCAGTGATTTC |
